# Refractoriness to subcutaneous implantable cardioverter defibrillator after frequent therapies for ventricular fibrillation storms in a Brugada syndrome case

**DOI:** 10.1186/s12872-020-01405-4

**Published:** 2020-03-13

**Authors:** Yasuhisa Nakao, Kazuyoshi Suenari, Kohei Yamashiro, Hiroshi Nakagawa, Nobuo Shiode

**Affiliations:** 1 Department of Cardiology, Hiroshima Citizens Hiroshima City Hospital, 7-33 Motomachi, Naka-ku, Hiroshima, 730-8515 Japan; 2 Department of Cardiology, Takatsuki General Hospital, Takatsuki, Japan; 3 Heart Rhythm Institute, University of Oklahoma Health Sciences Center, Oklahoma City, OK USA

**Keywords:** S-ICD, Brugada syndrome, Ventricular fibrillation, Defibrillation threshold

## Abstract

**Background:**

The subcutaneous implantable cardioverter defibrillator (S-ICD) is an alternative to the transvenous implantable cardioverter defibrillator for the prevention of sudden cardiac death. Here, we report a rare case of refractoriness to an S-ICD after frequent therapies for ventricular fibrillation (VF) storms.

**Case presentation:**

A 24-year-old man underwent a bout of syncope with vomiting and incontinence at home. He was brought to the emergency room and was witnessed to spontaneously go into VF successfully converted by external defibrillation. Previously, he was diagnosed with a type I Brugada electrocardiogram pattern by a pilsicainide administration test in another hospital. Although he had a family history of sudden cardiac death in 3 relatives, including his brother, he was followed closely without any therapies because he had never had an episode of syncope. He was implanted with an S-ICD without any trouble. Seven months later, frequent S-ICD shocks for VF storms occurred. His VF was controlled by using intravenous amiodarone, which was converted to an oral preparation. However, his VF recurred after another 2 months. The analysis of his S-ICD data revealed that 4 consecutive shock deliveries could not terminate his VF and the final shock delivered could fortunately terminate it because of a high defibrillation threshold test (DFT) due to an increasing shock impedance (64 to 90 Ω). First, we performed an epicardial Brugada syndrome ablation and subsequently replaced and repositioned the S-ICD lead from a left to a right parasternal site. After the re-implantation of the S-ICD, the DFT test improved to within normal range. According to the pathological analysis, infiltration of inflammatory cells and extensive fibrosis were confirmed in the subcutaneous tissue around the shock lead and S-ICD body.

**Conclusion:**

Frequent S-ICD shocks for VF storms might cause various pathological changes around the device and lead to a high DFT.

## Background

The subcutaneous implantable cardioverter defibrillator (S-ICD) is an alternative to the transvenous implantable cardioverter defibrillator for the prevention of sudden cardiac death. This could be useful for younger Brugada syndrome patients who do not need anti-tachycardia pacing [1]. However, the efficacy and safety of an S-ICD for ventricular fibrillation (VF) storms in Brugada syndrome patients are not well-known.

## Case presentation

**Fig. 1:**
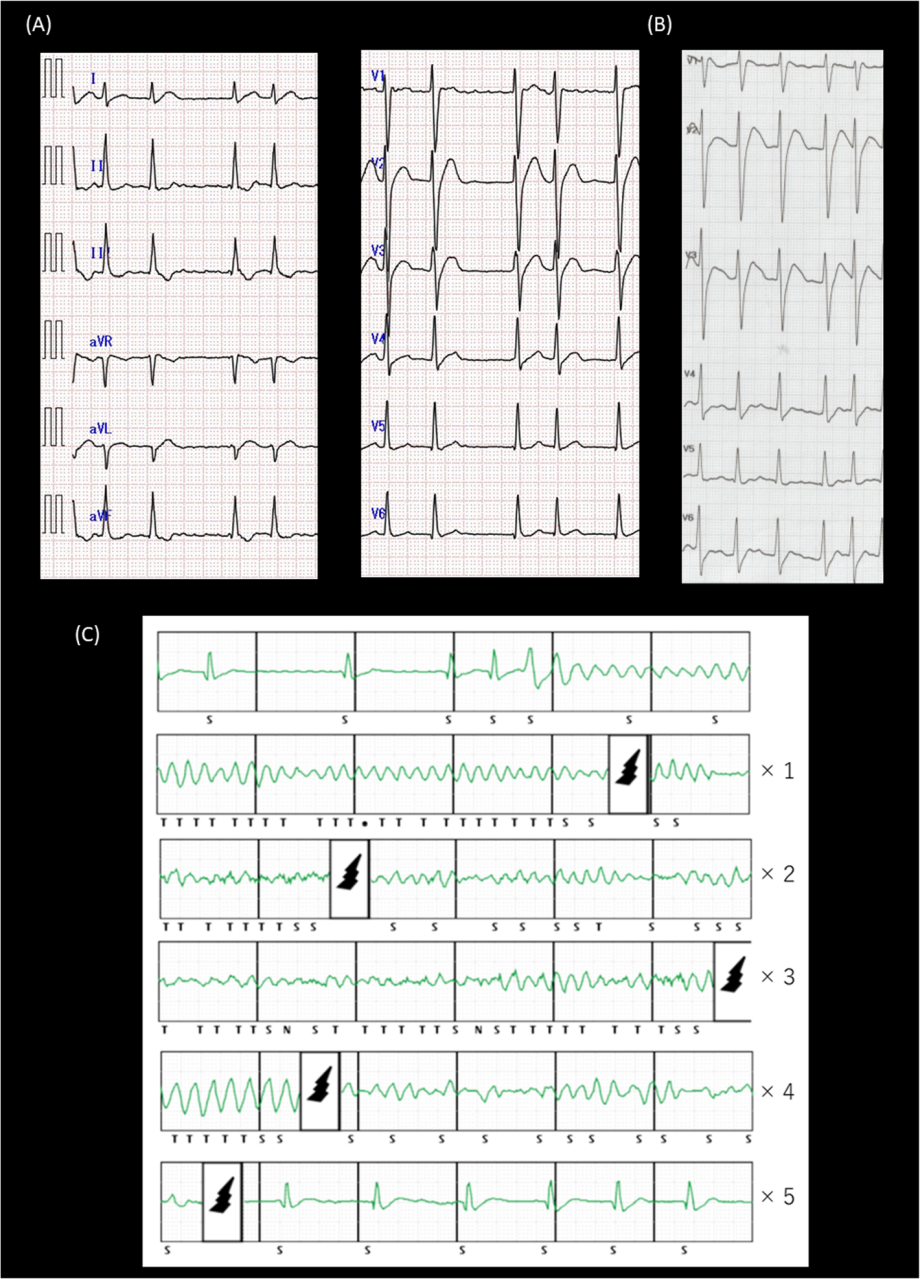
**a** The ECG exhibits atrial fibrillation on the standard 12 lead ECG. **b** The right sided pre-cordial leads at the 3rd intercostal space of the ECG exhibit a coved-type ST segment elevation. **c** After frequent S-ICD therapies for a VF storm, his VF recurred while waiting for a Brugada ablation. The analysis of his S-ICD data revealed that the final therapy terminated his VF

A 24-year-old man experienced a bout of syncope with vomiting and incontinence at home. After recovering consciousness, he called the emergency medical service by himself. He was brought to the emergency room and was witnessed to spontaneously go into ventricular fibrillation (VF) successfully converted by an external defibrillation. After the defibrillation, his ECG showed atrial fibrillation with a coved-type ST segment elevation recorded from the 3rd intercostal space (Fig. 1). Previously, he was diagnosed with a type I Brugada electrocardiogram pattern by a pilsicainide administration test in another hospital. Although he had a family history of sudden cardiac death in 3 relatives, including his brother, he was followed closely without any therapies because he had never had an episode of syncope. His physical examination was unremarkable. The cardiovascular examination revealed an irregular rhythm, with no pericardial friction rub, murmurs, carotid bruits, or jugular venous distention. In the evaluation of the patient, structural heart disease was ruled out by the results of exercise testing, chest roentgenography, echocardiography, and contrast-enhanced cardiovascular magnetic resonance imaging. In addition, ischemia and metabolic or electrolyte disturbances were ruled out by the laboratory test results. He was diagnosed with Brugada syndrome and was implanted with an S-ICD without any trouble. Ventricular fibrillation was induced and detected in the primary vector, which included the proximal sensing electrode and generator. Sinus rhythm was effectively restored via a sub-maximal 65 J shock. The device was programmed with a conditional zone of over 220 bpm and shock only zone of over 250 bpm.

**Fig. 2:**
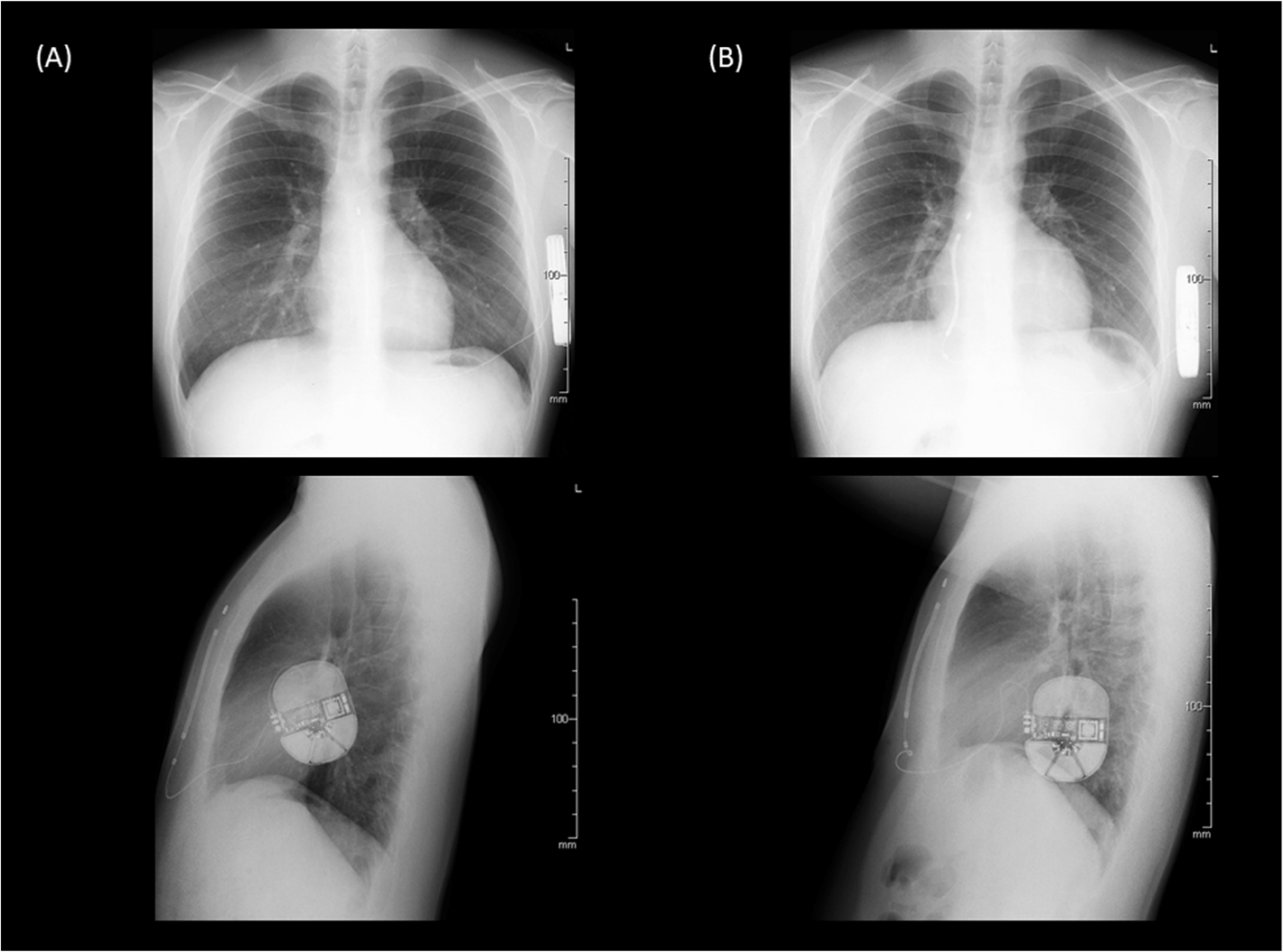
Chest X-ray (PA and RL views) before (**a**) and after (**b**) the re-implantation of the S-ICD system

**Fig. 3:**
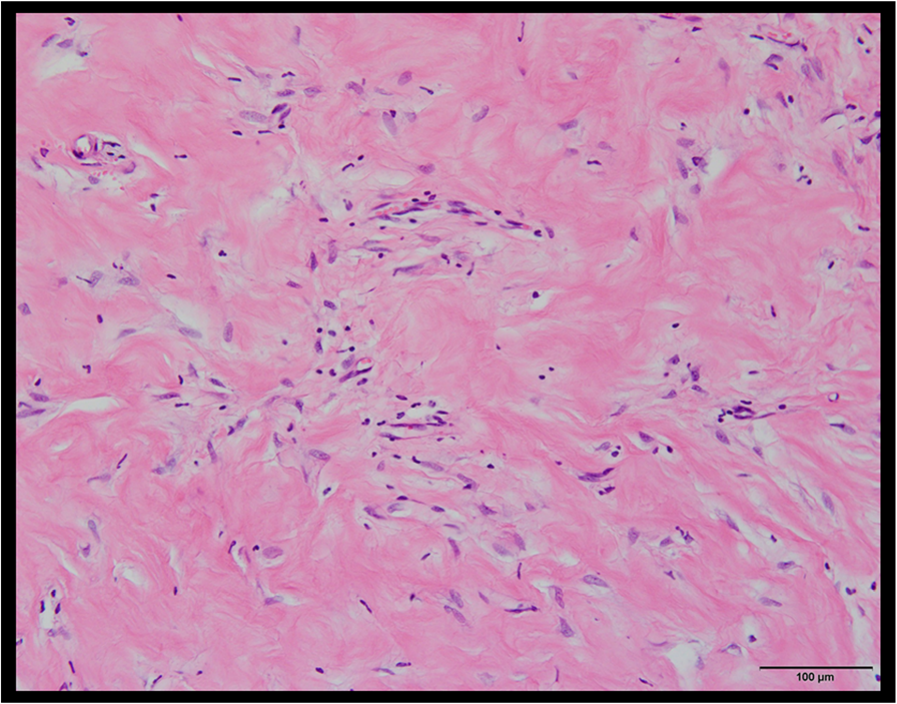
The pathological analysis of the subcutaneous tissue around the S-ICD could reveal infiltration of inflammatory cells and extensive fibrosis

Seven months later after the S-ICD implantation, he was transferred to our hospital due to a VF storm with 11 appropriate S-ICD therapies. He was effectively treated with an intravenous administration of amiodarone, which was subsequently converted to an oral preparation. We planned to perform an epicardial catheter ablation for Brugada syndrome. However, his VF recurred 3 weeks after the cessation of the amiodarone prior to the Brugada ablation. The analysis of his S-ICD data revealed that 4 consecutive shock deliveries could not terminate his VF, and the final shock delivered could fortunately terminate it (Fig. 2). Furthermore, a high defibrillation threshold (DFT) at that time was proven by an increasing shock impedance (64 to 90 Ω). First, we performed an epicardial Brugada syndrome ablation. Then, we replaced and repositioned the S-ICD can more infero-dorsally and the S-ICD lead from a left to right parasternal site. After the re-implantation of the S-ICD, the DFT and shock impedance improved to within the normal range (62 Ω). The device was received at the post Quality Assurance laboratory, and a thorough evaluation of the device was performed. The S-ICD device was exposed to simulated heart load conditions, and the defibrillation and sensing function were tested. The impedance testing was completed and all measurements were within normal limits. The device operated appropriately with no interruption in the therapy output at the programmed settings it was returned with. A series of electrical tests was also performed, and again, a normal device function was observed. This suggested that there were no abnormalities in the device itself. According to the pathological analysis, infiltration of inflammatory cells and extensive fibrosis were confirmed in the subcutaneous tissue around the S-ICD can (Fig. 3). Frequent S-ICD therapies for VF storms might cause various pathological changes around the device and lead to a high DFT.

## Discussion and conclusions

Brugada syndrome is an inherited arrhythmogenic disease, characterized by a coved-type ST-segment elevation in the right precordial ECG leads. In Brugada syndrome patients, the risk of sudden cardiac death increases due to VF. For patients with Brugada syndrome who have survived a sudden cardiac arrest, the HRS/EHRA/APHRS expert consensus statement recommends the implantation of an ICD rather than antiarrhythmic drug (AAD) therapy [2]. The S-ICD avoids important periprocedural and long-term complications associated with transvenous leads used with the conventional ICD [1]. This is beneficial for younger patients with Brugada syndrome who do not need anti-tachycardia pacing. The recent research from the EFFORTLESS S-ICD registry reported that a total of 104 patients had 278 appropriately treated VT or VF episodes, including 86 storm episodes [3]. Among them, storm events (86 episodes in 13 VT or VF storm events) were successfully converted for 12 events. Furthermore, the midterm performance rate of the conversion efficacy, complications, and inappropriate shocks by the S-ICD were comparable to the rates for the transvenous ICD [3]. In the present case, frequent electrical defibrillations for VF using the S-ICD, induced inflammation and fibrosis of the subcutaneous tissue and it was thought that the DFT increased. Recent research has reported that both the shock impedance and anatomic position of the S-ICD system (pulse generator and coil) are associated with the defibrillation efficacy [4]. As a fundamental improvement method, it was considered appropriate to change the position of the S-ICD. As a result of changing the position of the S-ICD, the shock impedance improved from 90 Ω to 62 Ω. Regarding the DFT test, the defibrillation using the S-ICD was successful the first time with a 65 J shock delivery. Although the previous research reported that obese patients (body mass index; BMI > 30 kg/m^2^) are at increased risk of a failed first S-ICD shock during defibrillation testing [5], his BMI was 24.6 kg/m^2^ (body weight: 63 kg, height: 160 cm).

This was considered to be due to changing the placement site of the S-ICD itself, not the rise in the defibrillation threshold due to the change in the body condition. The pathological change in the subcutaneous tissue around the S-ICD after frequent shock deliveries could also be supported. A previous report suggested that a higher shock impedance and high BMI are associated with failure of the standard 65 J DFT testing. Patients who fail the initial 65 J DFT have successful DFTs after reversing the shock polarity [6]. In this case, regardless of alternating the standard and reverse polarities, the last VF could not be terminated by 4 consecutive shock deliveries, and fortunately the VF terminated after the final shock delivery. The computer model suggests that a lateral-to-posterior generator placement with minimal fat underlying the coil and generator reduces the DFT with the S-ICD [7]. In this case, the first generator position was lateral-to-posterior and no change in the position was observed when the DFT increased. On the other hand, AAD therapy is considered in Brugada syndrome patients with an ICD who have recurrent arrhythmias resulting in ICD shocks. Quinidine, isoproterenol, and bepridil are well-known as useful AADs for the treatment of ventricular tachyarrhythmias in patients with Brugada syndrome [8–10]. In this case, since quinidine and bepridil were not effective for the VF storm and his ECG showed AF with a fast ventricular response at the time of admission, we initially treated him with an intravenous administration of amiodarone, which was effective for his VF storm. Although the amiodarone therapy could lead to an increased DFT, a previous study reported that the effect of amiodarone on the defibrillation energy requirements has been very limited [11]. Furthermore, in this case, his VF recurred due to stopping the amiodarone prior to the Brugada ablation. Our findings including the pathological analysis suggested that the frequent S-ICD therapies caused a critical inflammation and fibrosis of the subcutaneous tissue around the S-ICD can and lead to an increased DFT. Wakabayashi et al. reported the usefulness of lead repositioning from the left to the right sternal border if patients with an S-ICD have a high DFT during the implantation procedure [12]. To the best of the authors’ knowledge, this is the first report to present a Brugada syndrome case who required a device replacement due to a high DFT after frequent S-ICD therapies for VF storms.

In conclusion, frequent S-ICD therapies due to a VF storm might cause inflammatory changes with scarring in the subcutaneous tissue around the S-ICD system and that lead to the increased DFT in the present case.

## Data Availability

The datasets used in the case are available from the corresponding author upon reasonable request.

## Abbreviations

AAD: Antiarrhythmic drug
DFT: Defibrillation threshold
S-ICD: Subcutaneous implantable cardioverter defibrillator
VF: Ventricular fibrillation
